# Systems biological assessment of human immunity to BNT162b2 mRNA vaccination

**DOI:** 10.21203/rs.3.rs-438662/v1

**Published:** 2021-04-22

**Authors:** Prabhu S. Arunachalam, Madeleine K. D. Scott, Thomas Hagan, Chunfeng Li, Yupeng Feng, Florian Wimmers, Lilit Grigoryan, Meera Trisal, Venkata Viswanadh Edara, Lilin Lai, Sarah Esther Chang, Allan Feng, Shaurya Dhingra, Mihir Shah, Allie Skye Lee, Sharon Chinthrajah, Tina Sindher, Vamsee Mallajosyula, Fei Gao, Natalia Sigal, Sangeeta Kowli, Sheena Gupta, Kathryn Pellegrini, Gregory Tharp, Sofia Maysel-Auslender, Steven Bosinger, Holden T. Maecker, Scott D. Boyd, Mark M. Davis, Paul J. Utz, Mehul S. Suthar, Purvesh Khatri, Kari C. Nadeau, Bali Pulendran

**Affiliations:** 1 Institute for Immunity, Transplantation and Infection, Stanford University, Stanford, CA, USA.; 2 Center for Biomedical Informatics, Department of Medicine, Stanford University School of Medicine, Stanford, CA 94305, USA.; 3 Division of Infectious Diseases, Cincinnati Children’s Hospital Medical Center, Cincinnati, OH, USA.; 4 Department of Pediatrics, University of Cincinnati College of Medicine, Cincinnati, OH, USA.; 5 Yerkes National Primate Research Center, Atlanta, Georgia, USA.; 6 Department of Medicine, Division of Immunology and Rheumatology, Stanford University School of Medicine, Stanford, CA, USA.; 7 Sean N. Parker Center for Allergy & Asthma Research, Stanford, CA, USA.; 8 Department of Pathology, Stanford University School of Medicine, Stanford University, Stanford, CA, USA.; 9 Department of Microbiology and Immunology, Stanford University School of Medicine, Stanford University, Stanford, CA, USA.; 10 Howard Hughes Medical Institute, Stanford University, Stanford, CA, USA.; 11 Department of Medicine, Division of Pulmonary, Allergy and Critical Care Medicine, Stanford, CA, USA.

## Abstract

The emergency use authorization of two COVID-19 mRNA vaccines in less than a year since the emergence of SARS-CoV-2 represents a landmark in vaccinology^1,2^. Yet, how mRNA vaccines stimulate the immune system to elicit protective immune responses is unknown. Here we used a systems biological approach to comprehensively profile the innate and adaptive immune responses of 56 healthy volunteers vaccinated with the Pfizer-BioNTech mRNA vaccine. Vaccination resulted in robust production of neutralizing antibodies (nAbs) against the parent strain and a variant of concern, B.1.351, and significant increases in antigen-specific polyfunctional CD4 and CD8 T cells after the second dose. There was also a robust innate response induced within the first 2 days of the booster vaccination, compared to the first dose. Specifically, there were strongly enhanced: (i) frequency of CD14^+^CD16^+^ inflammatory monocytes; (ii) concentration of IFN-γ in the plasma, which correlated with enhanced pSTAT3 and pSTAT1 levels in monocytes and T cells; and (iii) transcriptional signatures of innate responses characteristic of antiviral responses, within 2 days following booster vaccination, compared to the primary response. Consistent with these observations, single-cell transcriptomics analysis of 242,479 leukocytes demonstrated a ~100-fold increase in the frequency of a myeloid cell cluster containing monocytes and dendritic cells, enriched in interferon-response transcription factors (TFs) and reduced in AP-1 TFs, only after the second immunization. Finally, we identified distinct molecular pathways of innate activation that correlate with CD8 T cell and nAb responses, and identify an early monocyte-related signature that was associated with the breadth of the nAb response against the B1.351 variant strain. Collectively, these data provide insights into the cellular and molecular responses induced by mRNA vaccines and demonstrate their capacity to prime the immune system to mount a more potent innate immune response following booster immunization.

The Pfizer-BioNTech mRNA vaccine, BNT162b2, has been administered to millions of people worldwide, and demonstrated a 95% efficacy in preventing severe COVID-19 disease^1^. Yet, although the antibody and T cell responses induced by this vaccine have been characterized in humans^2,3^, little is known about the innate immune responses stimulated by this vaccine, or by mRNA vaccines in general. Systems based approaches provide a platform to comprehensively investigate the molecular and cellular networks driving innate and adaptive immune responses to vaccines and infections^4-6^. Here, we used systems tools to analyze immune responses in 56 healthy volunteers who received two doses of the BNT162b2 vaccine. The demographics and self-reported symptoms of all volunteers are shown in Extended Data Tables 1 and 2. Of note, a large proportion of volunteers reported having various mild side-effects such as muscle aches, fatigue, headache and chills after secondary vaccination (Extended Data Table 2).

## Antigen-specific antibody and T cell responses

**Extended Data Fig. 1:**
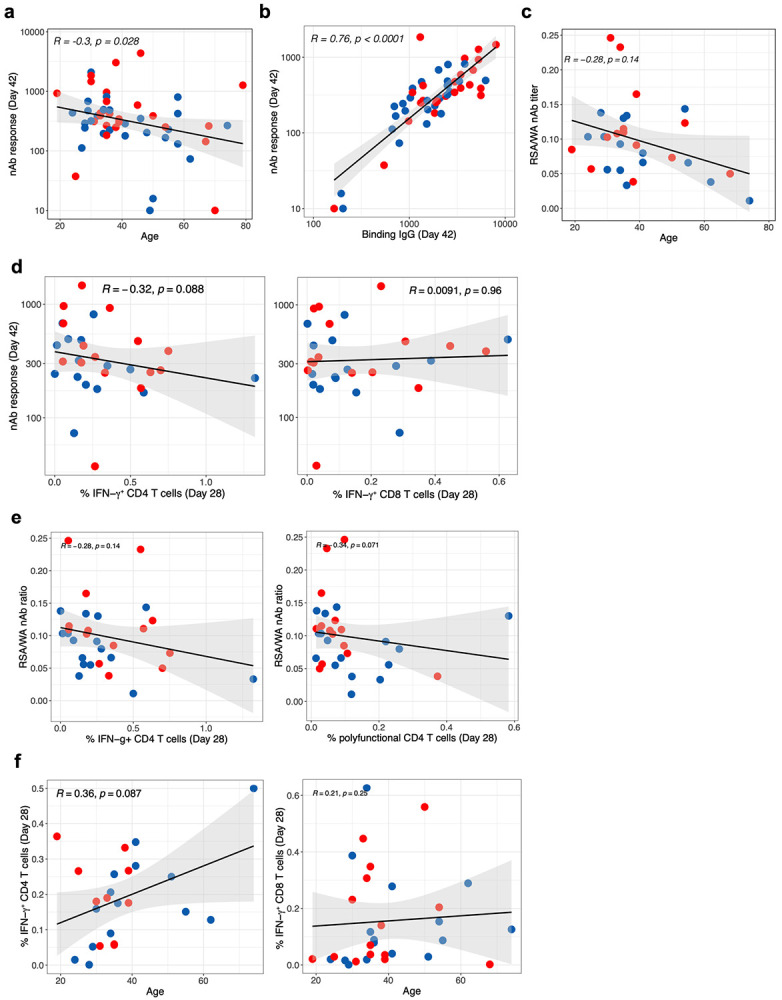
Correlation between antibody, T cell responses and age. **a – c,** Correlation between nAb responses and age (**a**), binding antibody and nAb titers (**b**), age and cross-neutralization index, ratio between nAb responses against B.1.351 to WA1 strains. **d,** Correlation between Spike-specific CD4 (left panel) and CD8 (right panel) T cell frequencies and nAb responses. **e,** Correlation between CD4 T cell frequencies, IFN-γ+ (left panel) or polyfunctional CD4 T cells expressing IL-2, IFN-γ and TNF-a (right panel). **f,** Correlation of Spike-specific CD4 (left panel) and CD8 (right panel) T cell responses with age. All correlations are two-sided Spearman’s correlation.

**Fig. 1.**
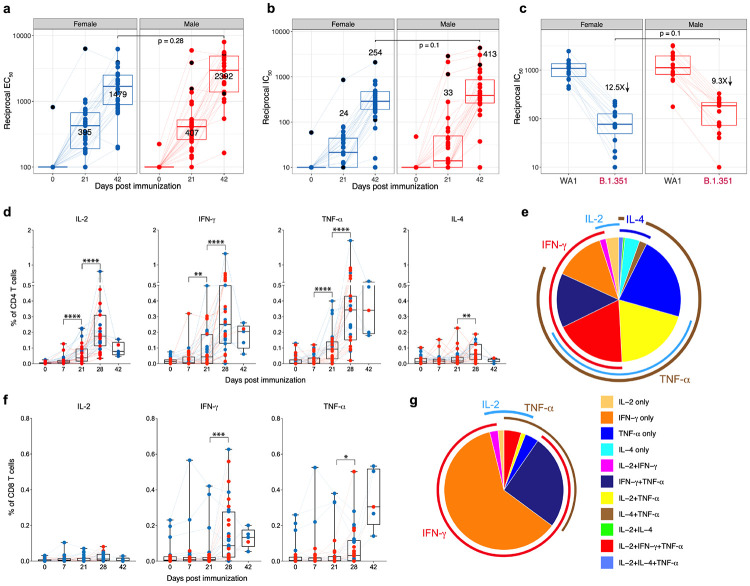
BNT162b2 vaccination induces robust antibody and T cell responses **a - b,** SARS-CoV-2 S-specific IgG titers in sera measured by ELISA **(a)** and authentic virus nAb titers measured by FRNT assay **(b)** at time points indicated on X-axis. The numbers within the plots represent GMT. **c,** Authentic virus nAb responses against the wild-type Washington (WA1) or B.1.351 variant of concern measured in sera collected on day 42. The numbers within the plots represent fold change between WA1 and B.1.351 strains. The statistical differences between groups were calculated using two-sided Mann-Whitney rank-sum test. Each dot represents a participant (N = 56). **d, f,** Spike-specific CD4 **(d)** and CD8 **(f)** T cell responses measured in blood at time points indicated on the X-axis. The statistical difference between time points within each group is calculated using two-sided Wilcoxon matched-pairs signed-rank test (*p < 0.05, **p < 0.01, ***p < 0.001 and **** p < 0.0001). Each dot represents a participant. We analyzed 31 participants at baseline, days 7, 21 and 28 and five participants at day 42. **e, g,** Polyfunctional profiles of CD4 **(e)** and CD8 **(g)** T cells. The pie charts represent the proportion of CD4 **(e)** and CD8 **(g)** T cells expressing one, two or three cytokines as shown in the legend. Blue and red dots indicate female and male participants, respectively.

We measured binding and neutralizing antibody (nAb) responses in sera collected at baseline, day 21 and day 42 post vaccination. All but three individuals showed detectable binding antibody (bAb) and nAb responses after the first dose, which was further boosted by ~5-fold and ~10-fold, respectively, after the secondary immunization (Fig. 1a, b). There was no significant difference in the magnitude of bAb or nAb responses between males and females (Fig. 1a, b). However, there was a modest inverse correlation of nAb responses with age (Extended Data Fig. 1a). Furthermore, bAb responses strongly correlated with nAb responses (Extended Data Fig. 1b). Four volunteers had a prior confirmed SARS-CoV-2 infection, of which three had undetectable baseline bAb and nAb responses (filled black circles in Fig. 1a, b). However, two of them and the fourth volunteer who showed detectable baseline titers increased >30-fold higher than the GMT (Geometric Mean Titers) of the rest of the volunteers after one immunization, and did not increase further after the boost, consistent with recent observations^7^. Notably, one participant who had a mild-moderate COVID-19 eight months prior to vaccination responded poorly even after two doses. We also measured nAb response against the variant of concern (VOC) B.1.351 using a live-virus neutralization assay in a subset of 30 participants. Consistent with previous studies^8^, there was an nAb response against B.1.351, with a marked (~10-fold) reduction in comparison to the WA1 parent strain (Fig. 1c). The cross-neutralization potential, measured as a ratio of nAb response between B.1.351 and WA1 strains, also showed a modest but statistically insignificant negative association with age (Extended Data Fig. 1c).

Vaccination also stimulated Spike-specific CD4 and CD8 T cell responses, more readily detectable 7 days after the secondary immunization (Fig. 1d, f). Consistent with the previous study^3^, the CD4 T cell responses were primarily Th1-type expressing IL-2, IFN-γ or TNF-a although there were low levels of IL-4 induction (Fig. 1e). On the other hand, IFN- γ and TNF-a were the dominant responses in CD8 T cells with 25 of the 31 participants responding with IFN- γ response atleast 3 times higher than the baseline (Fig. 1f, g). Of note, three individuals with no known exposure to SARS-CoV-2 or clinical symptoms associated with COVID-19 demonstrated ~0.2% of Spike-specific CD8 T cell responses at the baseline consistent with previous studies indicating that 10% of healthy individuals have cross-reactive CD8 T cell responses^9^. There was no significant correlation between T cell response and age or nAbs against the parent or B.1.351 strains (Extended Data Fig. 1b - d).

## BNT162b2 vaccination does not cause new onset autoantibodies or anti-cytokine antibodies (ACA).

**Extended Data Fig. 2:**
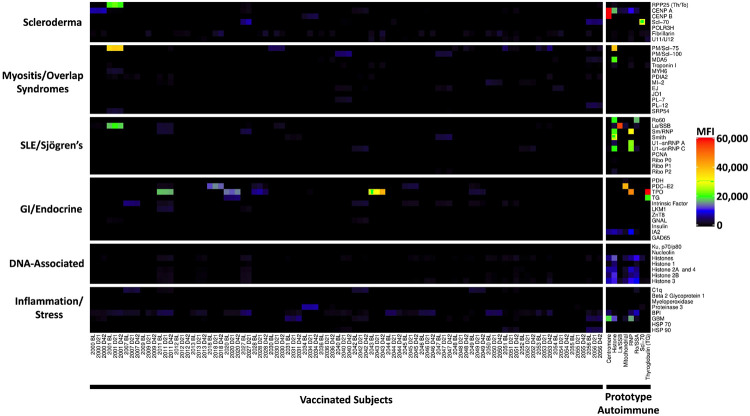
Autoantibodies in vaccinated subjects. Heatmap depicting serum IgG antibodies discovered using a 55-plex bead-based protein array containing the indicated autoantigens (Y-axis). Autoantigens are grouped based on disease (e.g., scleroderma, myositis and overlap syndromes such as mixed connective tissue disease (MCTD), SLE/Sjögren’s, and gastrointestinal and endocrine disorders), DNA-associated antigens, and antigens associated with tissue inflammation or stress responses. Vaccinated subjects are shown in the left panel (n=30 subjects, on day 0, day 21, and day 42 and n=1 on day 0 and day 21), and eight prototype autoimmune disorders are shown on the (right panel). Colors correspond to the MFI values shown at far right.

**Extended Data Fig. 3:**
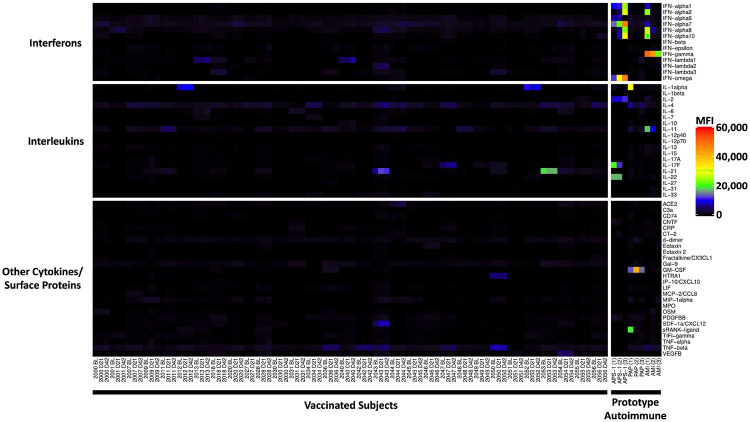
Anti-cytokine antibodies in vaccinated subjects. Heatmap using a 58-plex array of cytokines, chemokines, growth factors, and receptors. Cytokines are grouped on the y-axis by category (interferons, interleukins, and other cytokines/growth factors/receptors), while serum samples are shown on the x-axis. Vaccinated subjects are shown in the left panel (n=30 subjects, on day 0, day 21, and day 42 and n=1 on day 0 and day 21). Prototype samples from patients with immunodeficiency disorders are shown in the right panel and include three patients with Atypical Mycobacterial Infections (AMI), three patients with Pulmonary Alveolar Proteinosis (PAP), and three patients with Autoimmune Polyendocrine Syndrome Type 1 (APS1). Colors correspond to the MFI values shown at far right.

**Extended Data Fig. 4:**
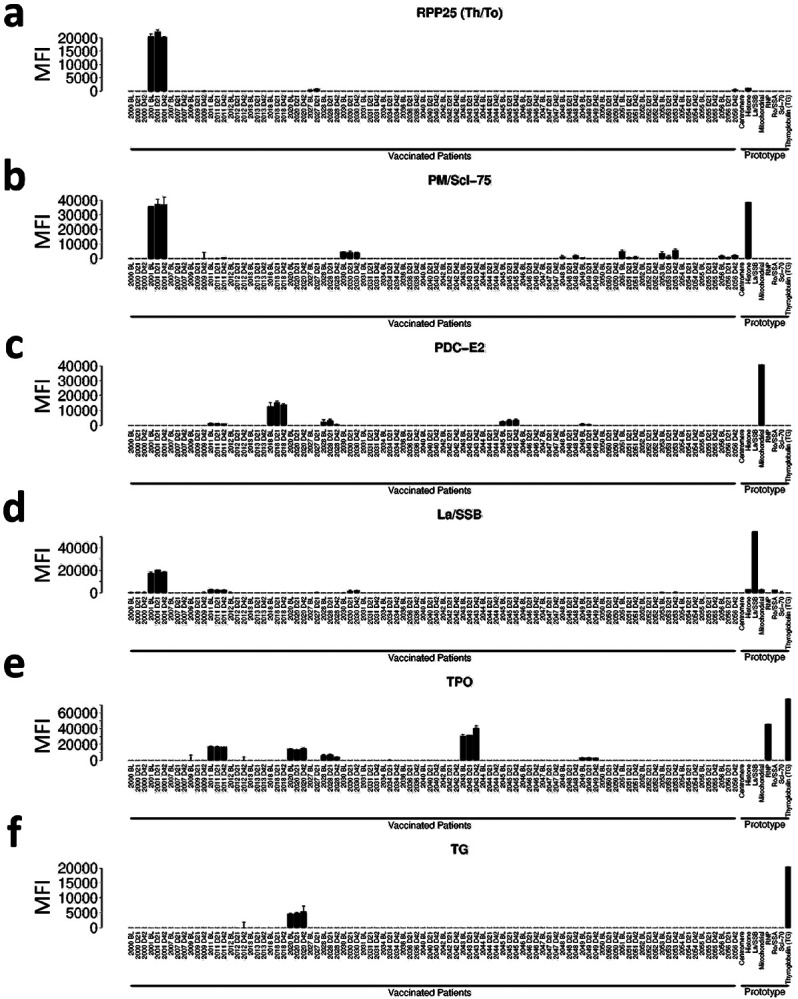
Pre-existing autoantibodies do not change in vaccinated subjects. Bar plots are shown for representative patients with high baseline MFI autoantibodies for select antigens (a-f). a. Anti-RPP25 (Th/To); b. Anti-PM/Scl-75; c. Anti-SSB/La; d. Anti-PDC-E2; e. Anti-Thyroperoxidase, TPO; f. Anti-Thyroglobulin, TG.

**Extended Data Fig. 5:**
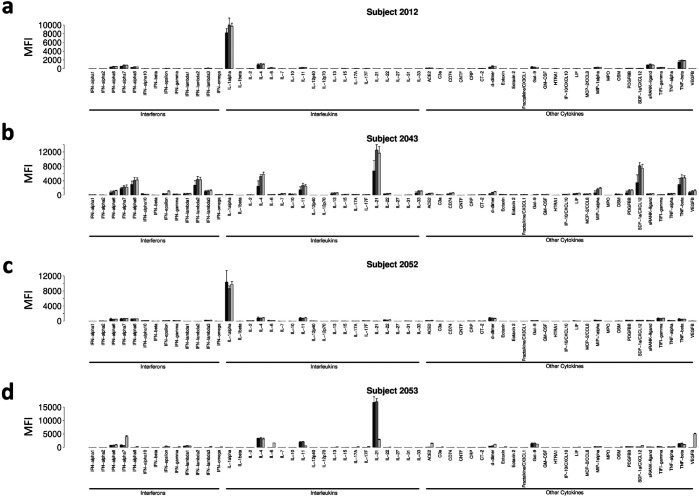
Pre-existing ACA do not change in vaccinated subjects. Bar plots are shown for representative patients with high baseline MFI ACA. Group bars represent antigens for baseline (black), D21 (grey), and D42 (white) timepoints. a. Subject 2012 ACA measurements; b. Subject 2043 ACA measurements; c. Subject 2052 ACA measurements; d. Subject 2053 ACA measurements.

Multiple studies have demonstrated the presence of serum autoantibodies^10 11,12 13 14^ and ACA^5 15^ in patients infected with SARS-CoV-2, as well as development of new-onset antibodies in a subset of hospitalized COVID-19 patients^16^. We screened serum samples from 31 participants and compared mean fluorescence intensity (MFI) of IgG autoantibodies and ACA on days 0, 21, and 42 using a 55-plex antigen array and a 58-plex cytokine array. We included prototype serum samples from 17 patients with autoimmune and immunodeficiency disorders as positive controls. Five vaccinated subjects had preexisting autoantibodies (three suggestive of autoimmune thyroiditis, one low level PDC-E2+ associated with primary biliary cirrhosis, and one subject positive for connective tissue disease antigens RPP25 (Th/To), PM/Scl75, and SSB (La), (Extended Data Fig. 2). Anti-cytokine antibodies were largely absent or were observed at low MFI (Extended Data Fig. 3). Two subjects, including one who was also TPO+, had anti-IL-21 autoantibodies, and two additional subjects had anti-IL-1 antibodies (Extended Data Fig. 4, 5). Importantly, in subjects with pre-existing autoantibodies or ACA, none had adverse events, nor did levels of pre-existing autoantibodies or ACA change in response to vaccination. New-onset autoantibodies or ACA were not observed in any of the vaccinated subjects (Extended Data Fig. 2, 3).

## Innate immune responses to mRNA vaccination

**Extended Data Fig. 6:**
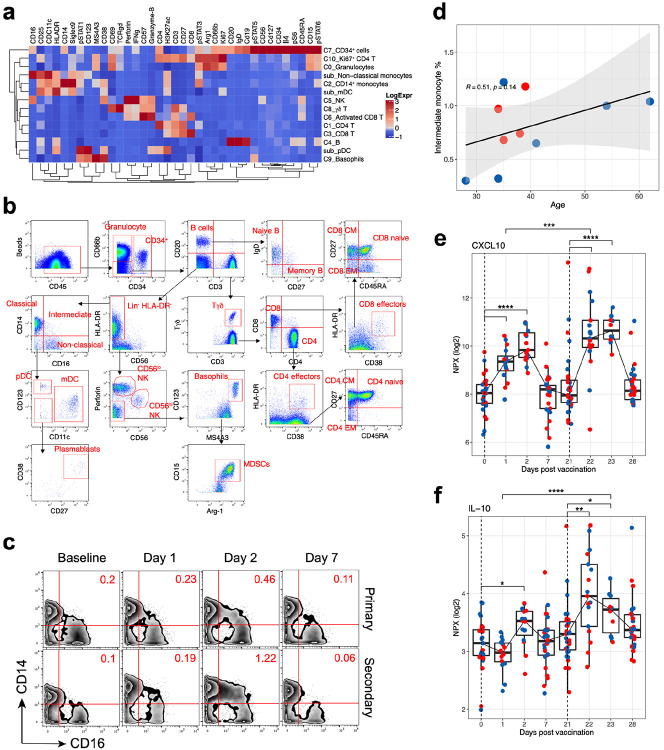
Innate immune responses to BNT162b2 vaccination. **a,** Heatmap showing expression of markers in the different cell clusters identified in the CyTOF dataset. **b,** Gating strategy identifying immune cell populations by the CyTOF panel. **c,** Mass cytometry plots showing CD14/CD16 expression of Lin^−^ HLA-DR^+^ population. **d,** Scatter plot showing two-sided Spearman’s correlation between frequency of CD14^+^ CD16^+^ intermediate monocytes on day 23 and age. **e – f,** Plasma levels of CXCL-10 (**d**) and IL-10 (**e**) determined by Olink. The statistical differences were analyzed using two-sided Wilcoxon matched-pairs signed-rank test or two-sided Mann-Whitney rank-sum test (*p < 0.05, **p < 0.01, ***p < 0.001 and ****p < 0.0001). Blue and red dots indicate female and male participants, respectively.

**Fig. 2.**
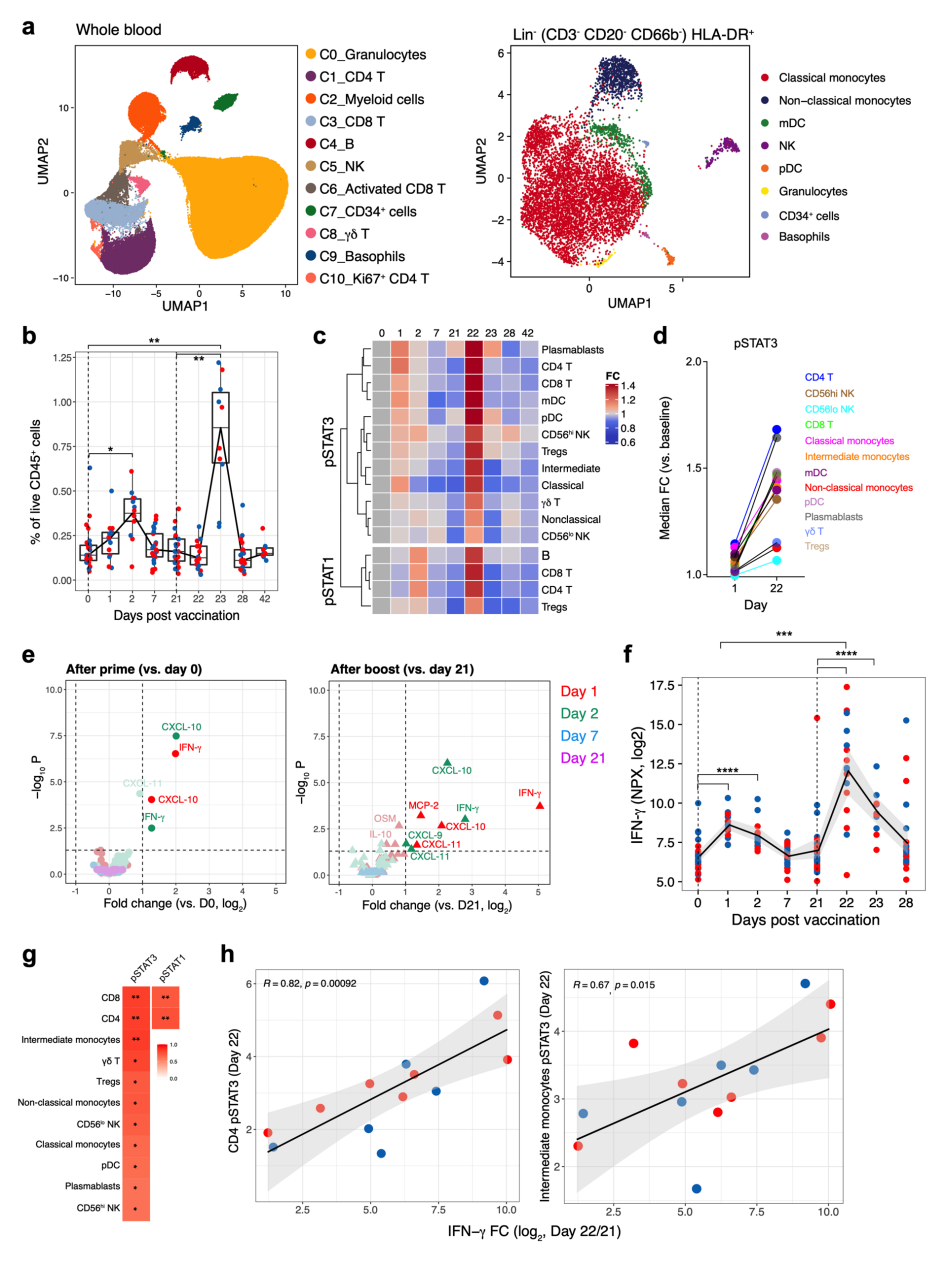
Innate immune responses induced by BNT162b2 vaccination. **a,** Representation of CyTOF-identified cell clusters in whole blood (or Lin^−^ (CD3^−^ CD20^−^ CD66b^−^) HLA-DR^+^ cells visualized by UMAP in two-dimensional space. **b,** Frequency of inflammatory monocytes (CD3^−^ CD20^−^ CD66b^−^ CD56^−^ HLA-DR^+^ CD14^+^ CD16^+^) as a proportion of live CD45+ cells at time points indicated on X-axis. **c,** A heatmap representing fold change (FC) of pSTAT3 and pSTAT1 levels in comparison to baseline in cell types indicated on Y-axis. These changes were statistically significant between the increase on day 1 after primary, and day 1 after secondary immunizations, as measured using two-sided Mann-Whitney rank-sum test (p < 0.05). **d,** FC in pSTAT3 levels in the indicated cell types on day 1 and day 22, compared to primary and secondary baselines, respectively. **e,** Volcano plots showing plasma cytokines significantly increased after primary (left panel) and secondary (right panel) vaccinations. **f,** Plasma IFN-γ levels after vaccination measured by Olink multiplex cytokine platform. **g,** Heatmap representation of two-sided Spearman’s correlation between increase in plasma IFN-γ on day 1 post-secondary vaccination and pSTAT3 or pSTAT1 levels in different cell types. The p-values were corrected for multiple testing. **h,** Scatter plots showing spearman’s correlation between pSTAT3 levels in CD4 T cells (left panel) and inflammatory monocytes (right panel) and plasma IFN-γ levels. In **b** and **f**, the statistically significant differences between the peak and baseline time points were measured using two-sided Wilcoxon matched-pairs signed-rank test. The difference peak time points were measuring using two-sided Mann-Whitney rank-sum test (*p < 0.05, **p < 0.01, ***p < 0.001 and **** p < 0.0001). Blue and red dots indicate female and male participants, respectively.

While previous studies have analyzed adaptive immune responses to BNT162b2 vaccination^3,17^, little is known about the innate immune responses induced by BNT162b2. To analyze innate immune responses, we first assessed whole blood samples of 27 individuals collected across multiple time points after vaccination using a 38-parameter mass cytometry (Cytometry by Time of Flight, CyTOF) panel containing an assortment of cytokines and phospho signaling markers (Extended Data Table 3). Unsupervised clustering identified 14 major cell types (Fig. 2a and Extended Data Fig. 6a) which were further subtyped manually (Extended Data Fig. 6b). The frequency of intermediate monocytes (CD14^+^CD16^+^ monocytes), key orchestrators of innate immunity, significantly increased 2 days after the first immunization. Strikingly, the frequencies were substantially higher on day 23, 2 days post-secondary vaccination, compared to the frequencies at day 2 post primary vaccination (Fig. 2b, and Extended Data Fig. 6c). There was no correlation with age (Extended Data Fig. 6d). In addition, the CyTOF analysis revealed greatly enhanced phosphoSTAT3 (pSTAT3) and phosphoSTAT1 (pSTAT1) in multiple cell types on day 1 after secondary immunization, relative to their modestly increased expression on day 1 post primary immunization (Fig. 2c, d). These data suggested that the BNT162b2 mRNA vaccination induced a heightened innate immune response following secondary immunization, relative to the response after the primary immunization.

To further investigate this phenomenon, we measured 92 different cytokines in plasma collected at various time points from 31 vaccinees using the Olink platform. Of the 67 cytokines that were detected within the dynamic range of the assay, the concentration of two cytokines, IFN-γ and CXCL-10, were significantly increased on days 1 and 2 after primary immunization (Fig. 2e, left panel). Similar to the observations on intermediate monocytes and pSTAT3 signaling, the concentrations of these cytokines were increased even further after the secondary immunization (Fig. 2e, right panel). IFN-γ in particular rose 11.3-fold between day 1 and 22 (Fig. 2f). CXCL-10, on the other hand, peaked on day 2 suggesting a response driven by IFN- γ (Extended Data Fig. 6d). Interestingly, the anti-inflammatory cytokine IL-10 also showed a similar pattern of response to that of IFN- γ although this trend did not reach statistical significance (Extended Data Fig. 6e). We also measured type I IFN (IFN-α and IFN-β) using ELISA, which were below detection limit (<2 pg/ml) at any time point after vaccination. Furthermore, there was a strong correlation between plasma IFN-γ levels and pSTAT1/3 expression levels across several cell types (Fig. 2g, h). Of note, the concentration of cytokines returned to baseline levels by day 28 (i.e., 7 days post-secondary vaccination), suggesting origin from an innate immune cell type. Collectively, these data demonstrate that vaccination with BNT162b2 stimulates low levels of innate immune responses after primary immunization, which strikingly increase after the secondary immunization.

## Transcriptional signatures induced by vaccination

**Extended Data Fig. 7:**
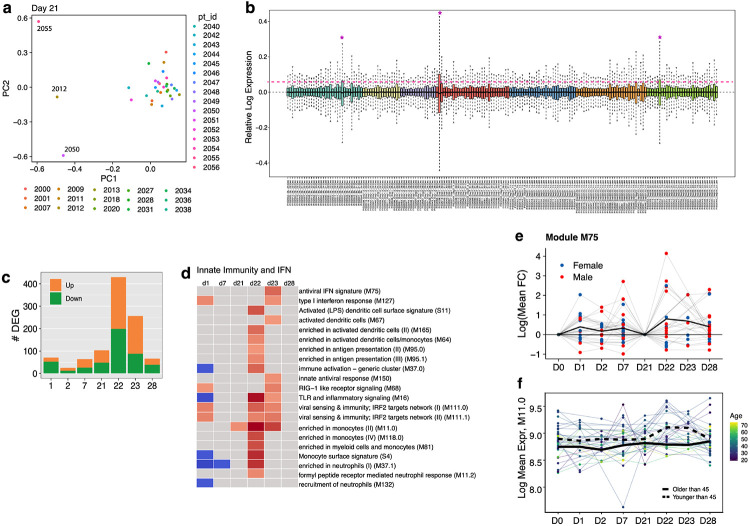
Transcriptomic signatures induced by BNT162b2 vaccination. **a,** PCA analysis of bulk RNAseq samples. **b,** RLE analysis of bulk RNAseq data. **c,** Number of genes differentially expressed (absolute log_2_ fold-change > 0.2 and Wald p < 0.01) at each timepoint. All time points were compared to universal baseline, day 0. Number of upregulated and downregulated genes are shown in orange and green, respectively. **d,** BTMs that were significantly enriched (false discovery rate [FDR] < 0.05, absolute normalized enrichment score [NES] > 2) after vaccination. GSEA was used to identify increased (red) or decreased (blue) enrichment of BTMs within gene lists ranked by Wald statistic between pre- and post-vaccination at each timepoint. **e - f,** Temporal expression patterns of genes within modules M75 (**e**) or M11 (**f**). Black lines represent the median fold change of gene expression across participants.

**Fig. 3.**
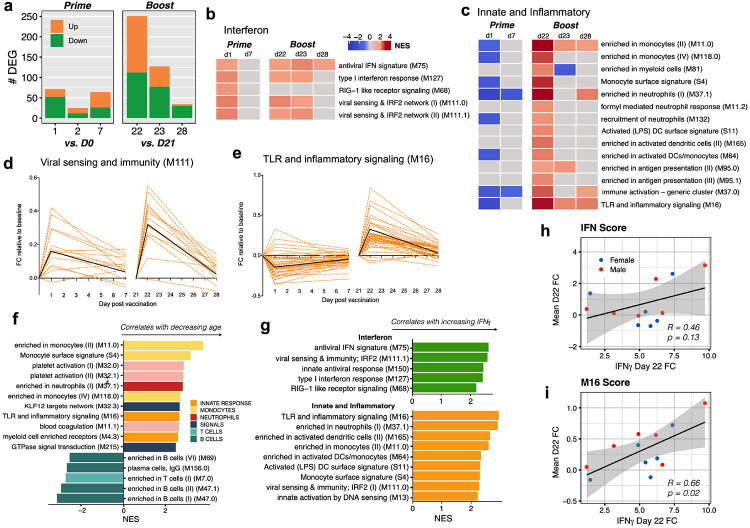
Transcriptional signatures induced by BNT162b2 vaccination. **a,** Number of genes differentially expressed (absolute log_2_ fold-change > 0.2 and Wald p < 0.01) at each timepoint. Days 1, 2, and 7 were compared against day 0; days 22, 23, and 28 were compared against day 21. Number of upregulated and downregulated genes are shown in orange and green, respectively. **b - c,** Interferon (**b**) or innate (**c**) BTMs that were significantly enriched (false discovery rate [FDR] < 0.05, absolute normalized enrichment score [NES] > 2) after vaccination. GSEA was used to identify increased (red) or decreased (blue) enrichment of BTMs within gene lists ranked by Wald statistic between pre- and post-vaccination at each timepoint, using the baselines described in (**a**). **d - e,** Temporal expression patterns of genes within modules M111.0 (**d**) or M16 (**e**). Black lines represent the median fold change of all genes. **f**, BTMs on day 22 significantly associated with age. **g,** BTMs on day 22 significantly associated with the increase in IFN-γ. GSEA was used to identify enrichment of BTMs within gene lists ranked by correlation with participant age (**f**) or IFN-γ day 22/21-fold change **(g**). Modules shown are those with NES > 2 and FDR < 0.05. **h - i,** Scatter plots showing the Pearson correlation of the mean day 22/21-fold change of genes in interferon modules M127, M75, M150, M111.1 and M68 (**h**) or module M16 (**i**) with IFN-γ.

We next investigated the transcriptomic changes induced by BNT162b2 vaccination. We performed bulk mRNA sequencing of 185 samples obtained from 31 participants across 7 time points. Six of 185 samples did not pass quality control and were removed from the analysis (Extended Data Fig. 7a, b). Strikingly, secondary immunization generated a much greater transcriptional response compared to primary immunization, with nearly a four-fold increase of DEGs found at day 22 compared to day 1 (Figure 3a). This was consistent with the increased markers of innate immunity demonstrated after secondary immunization by both CyTOF and Olink (Figure 2b-h).

In order to explore the specific transcriptional pathways altered in response to mRNA-based vaccination, we performed gene set enrichment analysis (GSEA)^18^ using a set of previously defined blood transcriptional modules (BTMs)^19^ at each post-vaccination timepoint. Both doses of BNT162b2 induced upregulation of antiviral and interferon response modules, including M75, M127 and M111.0 (Figure 3b). However, booster immunization led to a significantly broader innate response. In addition to induction of antiviral pathways, the boost dose led to increases in dendritic cell activation and upregulation of TLR signaling, monocyte, and neutrophil modules on days 22-23, which were previously decreased post-prime (Figure 3c). We compared fold changes of the genes within these modules and found that many antiviral genes showed a greater increase following the boost dose (Figure 3d), and other inflammatory genes switched from downregulation to upregulation between prime and boost (Figure 3e). These results were consistent regardless of the baseline timepoint used (Extended Data Fig. 7c, d). A complete list of enriched BTMs is presented in Supplementary Table 1.

As mortality to COVID-19 is highest among the elderly and older populations are known to mount inferior responses to many vaccines^20,21^, an important question we sought to address was whether or not there were age-associated differences in response to mRNA vaccination. We correlated the per-person fold changes of each gene with participant age and used GSEA to identify age-dependent response pathways. On day 22, we observed that younger subjects tended to have greater changes in monocyte, inflammatory response, and platelet-related expression, whereas older subjects had increased response in B and T cell modules (Figure 3f).

Finally, given that our serum cytokine analysis revealed that IFN-γ responses were also significantly higher following booster immunization, we asked whether there was any association between the level of IFN-γ and the increased innate responses following the boost. Indeed, both interferon response and other inflammatory modules were significantly enriched by GSEA when using genes ranked by correlation with IFN-γ on day 22 (Figure 3g). Furthermore, the average fold changes of these modules also correlated with IFN-γ (Figure 3h), suggesting that IFN-γ may play an important role in driving enhanced innate and antiviral expression post-boost.

## Single-cell transcriptional response to BNT162b2 vaccination

**Extended Data Fig. 8:**
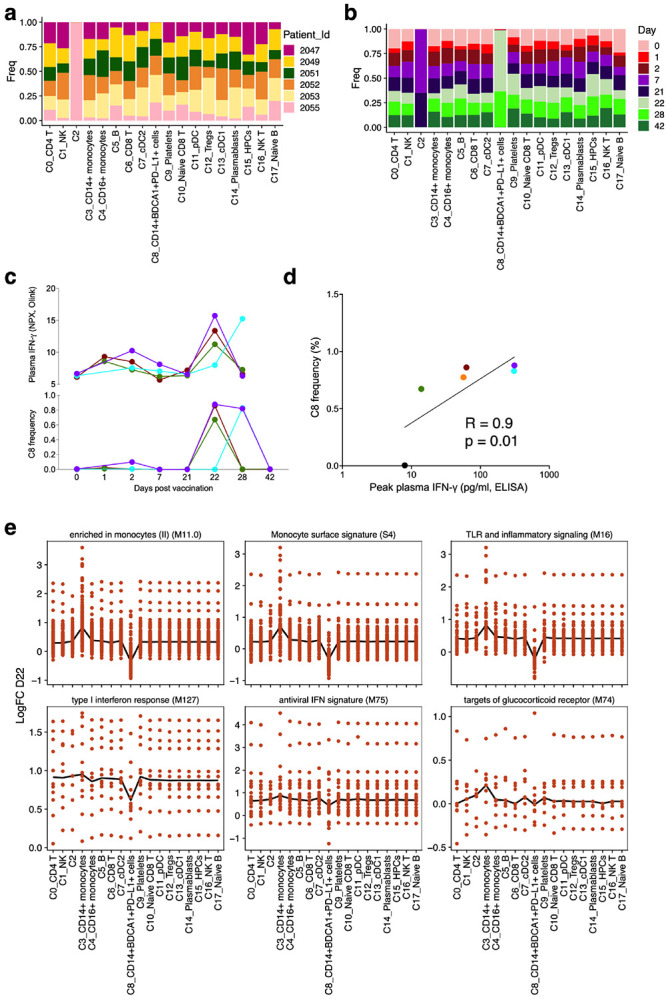
CITE-seq analysis of PBMCs. **a - b,** Fraction of cells in each cluster of CITE-seq data classified by subject (**a**) or time point (**b**), determined from all single cells that passed quality control. **c,** Frequency of C8 as a proportion of Lin- HLA-DR+ population or plasma IFN-γ levels as measured in Olink. **d,** Spearman’s correlation between plasma IFN-γ levels measured by ELISA and C8 frequency. **e,** Pseudobulk gene expression score showing the contribution from each cluster.

**Fig. 4.**
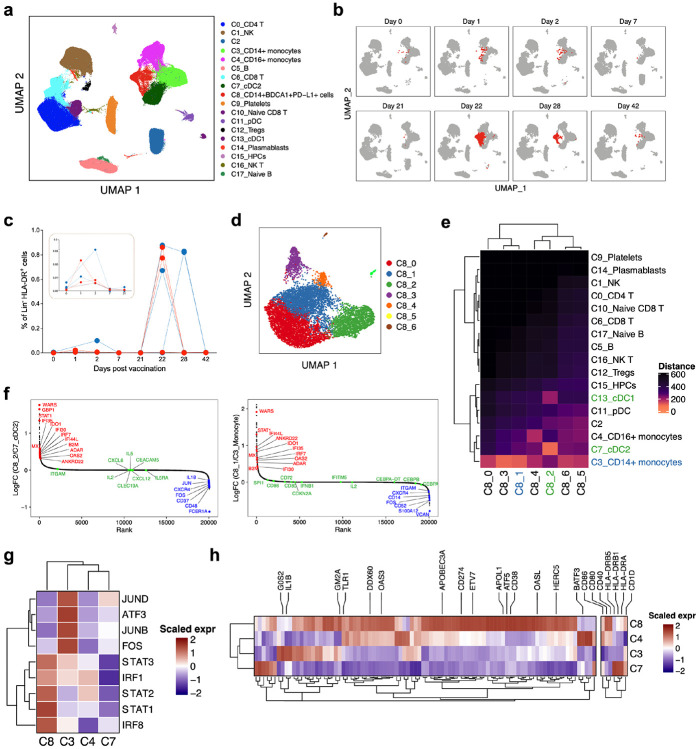
Single-cell transcriptional response to BNT162b2 vaccination. **a,** UMAP representation of cell types identified by single-cell transcriptional profiling of 242,479 PBMCs. **b,** Feature plots across time points showing cluster C8 in red and all the other cell types in grey. **c,** Frequency of cluster C8 as a proportion of Lin^−^ HLA-DR^+^ cell clusters (C3, C4, C7, C8, C11 and C13) in PBMCs identified by single-cell transcriptional profiling. **d,** UMAP representation of subclusters within C8 resolved using Louvain clustering. **e,** Heatmap showing Euclidean distance between subclusters within C8 and the rest of the cell types identified by single-cell profiling. **f,** Differentially expressed genes (DEGs) were determined between C8_2 or C8_1 and their closest cell clusters. The genes were ranked by FC between C8 and their parental clusters and plotted. Genes in red, green and blue fonts represent genes upregulated, unchanged or downregulated in C8 subcluster compared to its closest parental cluster. **g,** Heatmap showing scaled expression of key interferon-response and AP-1 transcription factors determined in the previous study by Wimmers, et al. 2021 in myeloid cell clusters in this study. **h,** Heatmap showing an extended set of genes that showed enhanced accessibility and increased expression on day 21/22 following H5N1/AS03 vaccination and activation markers in myeloid cell clusters. Blue and red dots indicate female and male participants, respectively.

We used cellular indexing of transcriptomes and epitopes by sequencing (CITE-seq) to determine the cellular origin of the enhanced antiviral and inflammatory gene signatures, and to more broadly characterize transcriptional signatures induced by vaccination at the single-cell level. To this end, we analyzed 45 PBMC samples from 6 individuals across seven time points (days 0, 1, 2, 7, 21, 22, 28 and 42). We used an “enrich-mix” strategy in which we enriched dendritic cells (DCs) and mixed with total PBMCs at a ratio of 1:2 to capture minor populations such as plasmacytoid dendritic cells (pDCs) sufficiently in the CITE-seq^4^. After preprocessing and quality control, we obtained 242,479 high quality transcriptomes, which were segregated into 18 cell clusters (Fig. 4a, Extended Data Fig. 8a, b). Strikingly, one cluster C8 (annotated C8_CD14^+^ BDCA1^+^ PD-L1^+^), expressing *CD14, VNN, CD1C, FCGRIA,* and *CD274* and other myeloid markers at gene and/or protein level, emerged on day 22, 1-day after secondary vaccination (Fig. 4b). These cells also expressed several ISGs including *IFI30, IFITM3, WARS* and *GBP1,* and constituted only ~0.01% of the Lin^−^ HLA-DR^+^ population on day 1 after primary immunization but increased almost 100X to ~1% one day after secondary immunization (Fig. 4c). Notably, the emergence of C8 correlated with plasma IFN-γ levels measured by Olink or an independent ELISA assay (performed because data of 2 participants were unavailable in Olink due to technical reasons), with the participant 2053 demonstrating a delayed increase in IFN-γ as well as C8 on day 28 (Extended Data Fig. 8c, d). Furthermore, iterative removal of each cluster from a pseudobulk score showed that C8 contributed to IFN and monocyte BTMs observed in the bulk transcriptomics data (Extended Data Fig. 8e). To further delineate the cellular composition of C8, we re-embedded C8 with UMAP, using harmony to correct for participant-specific biases. Using Louvain clustering, we resolved seven distinct clusters within the original C8 cluster (Fig. 4d). The cluster C8 proved to a heterogeneous mix of classical monocytes (C8_0, C8_1 and C8_3), cDC2 (C8_2) and intermediate monocytes (C8_4) as evidenced by the proximity to the original clusters measured by Euclidean distance (Fig. 4e). More interestingly, two sub clusters, C8_1 and C8_2, expressed significantly higher ISGs compared to their parent clusters (Fig. 4f). Additionally, they showed a reduced expression of AP-1 transcription factors *FOS* and *JUN*. Wimmers et al. recently described an epigenetically-remodeled monocyte population emerging in humans 21 days post vaccination with one dose of H5N1/AS03, and peaking 21 days post vaccination with two doses. The chromatin accessibility profile of these monocytes demonstrated an enhanced accessibility of ISGs and IRF/STAT transcription factors and reduced accessibility of AP-1 transcription factors, and more importantly, showed heightened resistance to infection with blood-borne viruses^22^. We asked if C8 represents an analogous cell type at the transcriptional level. We found that the C8 has a relatively higher expression of TFs *IRF1, STAT1, STAT2, STAT3, IRF8* and reduced levels of AP-1 TFs *FOS, JUNB, JUND* and *ATF3,* the same TFs that defined the monocyte population in the previous study (Fig. 4g). We also confirmed this using an extended set of genes for which the chromatin accessibility profile was higher 21 days after H5N1/AS03 vaccination (Fig. 4h).

**Fig. 5.**
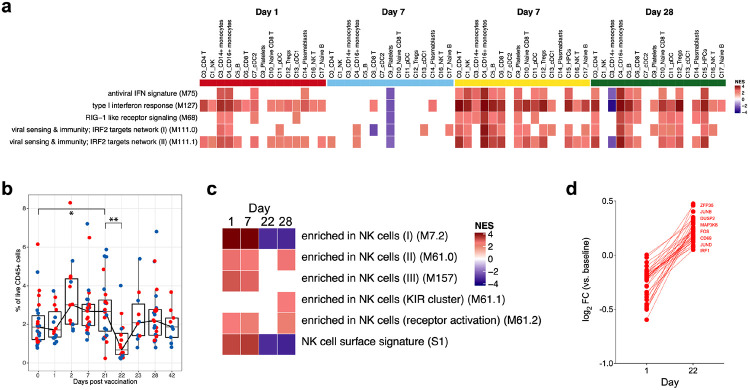
Broad transcriptional changes following BNT162b2 vaccination **a,** Significantly enriched interferon BTMs (false discovery rate [FDR] < 0.05, absolute normalized enrichment score [NES] > 2) across clusters over time. Days 1 and 7 were compared against day 0; days 22 and 28 were compared against day 21. GSEA was used to identify increased (red) or decreased (blue) enrichment of interferon BTMs. **b,** Frequency of NK cells as a proportion of live CD45^+^ cells in whole at time points indicated on X-axis. The statistical differences between time points were analyzed using two-sided Wilcoxon ranked-pairs. Blue and red dots indicate female and male participants, respectively. **c,** Significantly enriched NK cell modules (FDR < 0.05, absolute NES > 2) within the NK cell cluster. Timepoint comparisons and GSEA procedure were the same as in **a**. **d,** Log_2_ fold change of top 50 DEGs between NK cells from day 22 samples versus day 1 samples.

Next, we set out to identify transcriptional changes induced by vaccination more broadly within each cell type. Given that we observed a higher level of pSTAT1/3 in multiple cell types using CyTOF and a higher magnitude of IFN response after secondary immunization by bulk RNAseq, we asked if there is an enhanced IFN response in multiple cell types or it is primarily driven by the emergence of cluster C8. Interestingly, the IFN signature was induced in all cell types present on day 1 and day 22, and the higher magnitude of response on day 22 was more evident (Fig. 5a). The decrease in NK cell signatures on day 22 in bulk RNAseq was another intriguing feature observed in the bulk RNAseq. We have previously shown that the NK cells decrease in frequency following TIV vaccination, especially in young adults^23^. In line with this, we observed a significant reduction in the frequency of NK cells on day 22 in the CyTOF dataset (Fig. 5b). However, the genes in the NK cell modules were also significantly downregulated within NK cells present on day 22 (Fig. 5c). Conversely, the NK cells on day 22 had a higher activation status and higher levels of AP-1 transcription factors known to be driven by IL-2-mediated activation of NK cells^24^ (Fig. 5d).

## Comparison of transcriptional responses with other vaccines

**Fig. 6.**
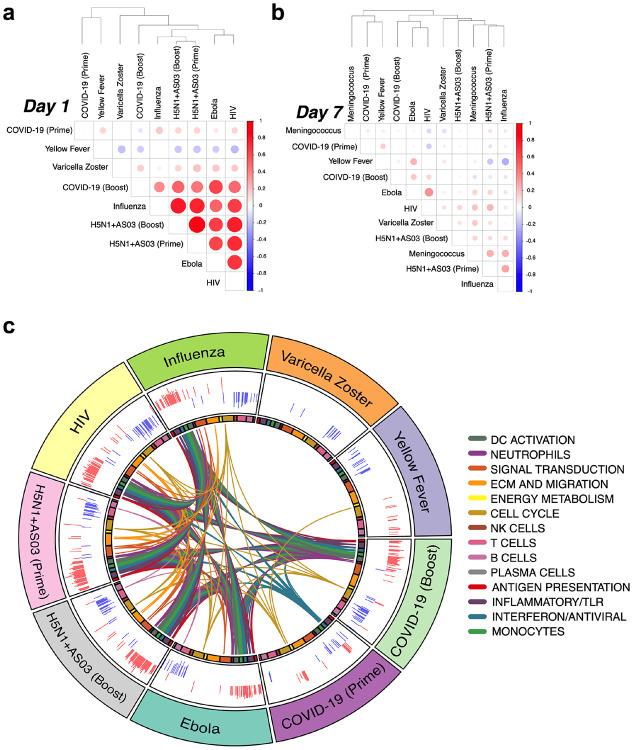
Comparison of transcriptional responses with other vaccines **a,** Correlation matrix of vaccines on day 1. Spearman correlation was computed using mean fold changes over all genes between each pair of vaccines. Circle size and color represents the correlation coefficient. **b,** Correlation matrix of vaccines on day 7. **c,** Circos plot of the overlap across vaccines in enriched BTMs on day 1. GSEA was performed on genes ranked by day 1 versus baseline t-statistic in each vaccine. Each segment of the circle represents one vaccine, and each point in a segment represents a single BTM. Bars in outer circle represent the NES of significantly enriched BTMs (FDR<0.05). Lines connect BTMs with a significant positive enrichment shared between vaccines. Inner circle boxes and line colors represent the functional groups of the BTMs.

As mRNA vaccines have only recently received approval for use in humans, the degree to which these vaccines induce similar or distinct immune responses compared to other vaccine types, such as inactivated or live attenuated vaccines, is unknown. To address this, we utilized a set of previously published vaccine trials from our group as well as several publicly available datasets to perform a comparative analysis with Pfizer-BioNTech BNT162b2 (see Extended Data Table. 4 for a summary of included vaccine datasets). In order to compare the relative similarity in transcriptional responses between vaccines, we generated similarity matrices through pairwise correlations of mean gene fold changes between vaccines at days 1 and 7 post-vaccination. While the day 1 response to the prime dose of BNT162b2 showed little overlap with other vaccines, the day 1 boost response was broadly similar to a group of vaccines containing either potent adjuvants (H5N1+AS03), live viral vectors (Ebola and HIV), or inducing a recall response (inactivated influenza) (Fig. 6a). Meanwhile, day 7 responses to both the prime and boost dose exhibited very weak correlation both between themselves and with other vaccines, suggesting little commonality in induced transcriptional signatures (Fig. 6b).

**Extended Data Fig. 9:**
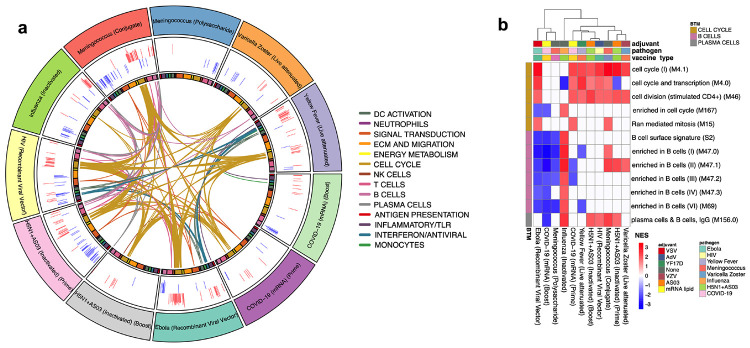
Comparison of transcriptional responses with other vaccines **a,** Circos plot of the overlap across vaccines in enriched BTMs on day 7. GSEA was run on genes ranked by day 7 versus baseline t-statistic in each vaccine. Each segment of the circle represents one vaccine, and each point in a segment represents a single BTM. Bars in outer circle represent the NES of significantly enriched BTMs (FDR<0.05). Lines connect BTMs with a significant positive enrichment shared between vaccines. Inner circle boxes and line colors represent the functional groups of the BTMs. **b,** Heatmap of cell cycle, B cell, and plasma cell BTMs on day 7. Cell cycle, B cell, and plasma cell BTMs that were significantly enriched (FDR<0.05) on day 7 after either dose of BNT162b2 are shown. Color represents significant NES.

To identify the specific pathways induced by BNT162b2 that were unique or shared with other vaccines, we performed BTM-level GSEA on each vaccine dataset using genes ranked by pre- versus post-vaccination t-statistic at each timepoint. We found that on day 1, the boost dose induced a robust innate response including upregulation of modules involved in antigen presentation, dendritic cell and monocyte activation, interferon signaling, and inflammatory responses, all of which were commonly induced by the Ebola and HIV live viral vector vaccines, seasonal influenza vaccine, and both doses of H5N1+AS03 (Fig. 4c). Conversely, the prime dose produced a much narrower response, only activating a limited set of interferon signaling modules. Instead, on day 7 the inverse trend occurred, with the boost dose having almost no commonly enriched modules but the prime dose sharing a cell cycle-related transcriptional signature with many vaccines (Extended Data Fig. 9a). However, in most other vaccines, the cell cycle signature is also associated with upregulation of B cell and plasma cell modules, reflecting the emergence and expansion of antibody-secreting cells^19,23^. This induction of B cell and plasma cell modules was absent in the BNT162b2 prime dose day 7 response (Extended Data Fig. 9b). Given that BNT162b2 successfully promoted a robust antibody response (Fig. 1), the lack of any detectable plasma cell or B cell signature on day 7, particularly post-boost, was surprising and unlike other vaccine responses that we are aware of. I. It is possible that the kinetics of the plasma cell response to this vaccine are more delayed and this signature was therefore not captured at the day 7 timepoint.

## Transcriptional correlates of antibody and T cell responses

**Fig. 7.**
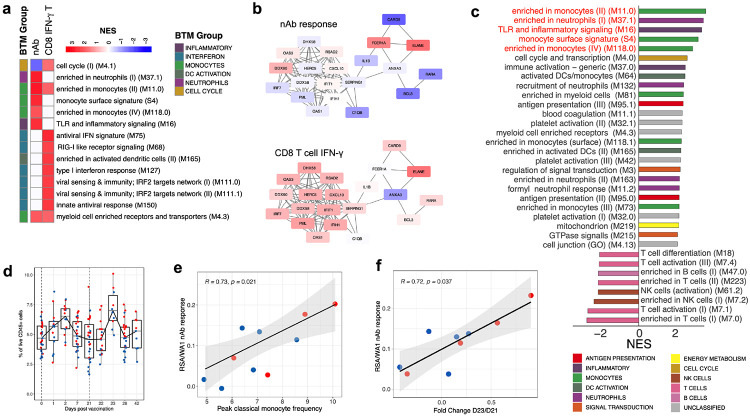
Transcriptional correlates of nAb and T cell responses **a,** BTMs associated with the nAb or CD8 IFNg T cell response to BNT162b2. GSEA was run using BTMs on gene lists ranked by correlation with either day 42 nAb titers or day 28 antigen-specific CD8+ IFN-γ+ T cell frequencies. Modules shown are those with NES > 2 and FDR < 0.05. **b,** Correlation of genes in M75, an antiviral BTM, on day 22 with the day 42 nAb response (top) or day 28 antigen-specific CD8+ IFN-γ+ T cell frequencies (bottom). Color represents the Pearson correlation coefficient. **c,** BTMs correlated with cross-neutralization index, ratio of B.1.351:WA1 nAb titers, analyzed as in (**a**). Modules shown are those with NES > 2 and FDR < 0.05. **d**, Frequency of classical monocytes (Lin^−^ HLA-DR^+^ CD14^+^ CD16^−^ cells) in whole blood samples analyzed by CyTOF. **e - f,** Scatter plots of two-sided Spearman’s correlation between cross-neutralization index and peak (day 23) classical monocyte frequency **(e)** or a gene score created in the bulk RNAseq data using the cluster-defining genes of the classical monocyte cluster, C3, in CITE-seq.

As cellular and humoral immunity are the chief functional components mediating protection from infection, a key question is whether early transcriptional signatures exist that are associated with either the antibody or T cell responses following vaccination with mRNA vaccines. We therefore used GSEA to identify transcriptional modules whose expression at various timepoints post-vaccination was correlated with either the day 42 nAb or day 28 CD8+ IFN-γ+ T cell response (Fig. 7a). In general, there was little overlap between signatures associated with the nAb and IFN-γ CD8 T cell response, suggesting that there are distinct molecular pathways leading to cellular and antibody responses to BNT162b2. On day 22 (1-day post-boost), there was a striking divergence in signatures, with monocyte-related modules correlated with nAb responses while interferon and antiviral signatures highly associated with the later day 28 IFN-γ CD8 T cell response (Fig. 7b). Surprisingly, plasma cell and cell cycle modules, which have previously been identified at day 7 as robust signatures of antibody response to other vaccines such as inactivated influenza vaccine^23^, were not associated at day 7 following either the prime or boost dose with the day 42 nAb titers.

The continued evolution of SARS-CoV-2 variants is a serious concern for the success of ongoing vaccination efforts. Therefore, we evaluated whether there are innate correlates of the cross-neutralization potential induced by vaccination. To this end, we defined a cross-neutralization index, using a ratio of variant to WA1 nAb titers, and used an enrichment approach to identify correlates of cross-neutralization. Monocyte and neutrophil BTMs as well as TLR and innate immune pathways were highly associated with cross-neutralization index (Fig. 7c) suggesting a central role for myeloid cells in the effective immunity induced by mRNA vaccination. More interestingly, the frequency of classical monocytes at peak, 2 days post-secondary vaccination, as measured by CyTOF, strongly correlated with cross-neutralization index (Fig. 7d). To further evaluate this, we determined a gene score that defines C3, the classical monocyte cluster in the CITE-seq data, and asked if this gene score in the bulk RNAseq also correlates with cross-neutralization. Clearly, there was a strong correlation (Fig. 7e). Collectively, these data demonstrate that while IFN signatures are associated with the CD8 T cell responses, monocyte and neutrophil gene signatures and TLR signaling BTMs strongly correlate with an nAb response against the WA1 as well as the B.1.351 strain.

In summary, our study describes a systems-based analysis that provides novel insights into the innate and adaptive immune responses to the Pfizer-BioNTech mRNA vaccine. We used a multiomics approach to define early transcriptional correlates of T cell and antibody responses. The data also demonstrate an enhanced innate immune response following secondary immunization indicating an innate memory-like response^25,26^. The mechanisms underlying the enhanced secondary innate response, and whether or not T and B cells provide feedback to stimulate an enhanced innate response, warrant further investigation. Importantly, our analysis of transcriptional signatures of BNT162b2 vaccination relative to those induced by six other vaccines provides a useful benchmark to contextualize mRNA vaccines with other vaccine types including live-viral vectors, recombinant viruses, adjuvanted and unadjuvanted subunit vaccines.

## Methods

### Human subjects and experimentation

Fifty-six healthy volunteers were recruited for the study under informed consent. The study was approved by Stanford University Institutional Review Board (IRB 8269) and was conducted within full compliance of Good Clinical Practice as per the Code of Federal Regulations. The demographics of all participants were provided in Extended Data Table 1.

### Anti-S binding ELISA

SARS-CoV-2 Spike protein was purchased from Sino Biologicals. 96-well high binding plates were coated with 100 ng of S protein diluted at a concentration of 2 μg/ml in PBS. Next morning, the plates were washed once, blocked with 3% non-fat milk in PBS containing 0.1% Tween 20 (PBST) for 1 h at room temperature. Sera samples serially diluted in 1% non-fat milk containing PBST was added to the plates and incubated at 37°C for 1 h. The plates were washed 3X with PBST, horseradish peroxidase conjugated goat anti-monkey IgG (γ-chain specific, Alpha Diagnostics, 1:4,000 dilution), in PBS-T containing 1% non-fat milk was added and incubated for 1 hour at RT. Wells were washed 3x with PBST before addition of 3,3',5,5'-Tetramethylbenzidine (TMB) substrate solution. The reaction was stopped after 12 minutes by addition of 0.16 M sulfuric or 1 M hydrochloric acid. The optical density (OD) at 450 nanometers was measured with a Biorad microplate reader.

### Focus Reduction Neutralization Titer assay

Neutralization assays with authentic SARS-CoV-2 virus were performed as previously described^27^. Sera samples were serially diluted (three-fold) in serum-free Dulbecco’s modified Eagle’s medium (DMEM) in duplicate wells and incubated with 100–200 FFU infectious clone derived SARS-CoV-2-mNG virus^28^ at 37 °C for 1 h. The Ab-virus mixture was added to VeroE6 cell (C1008, ATCC, #CRL-1586) monolayers seeded in 96-well blackout plates and incubated at 37 °C for 1 h. Post-incubation, the inoculum was removed and replaced with pre-warmed complete DMEM containing 0.85% methylcellulose. Plates were incubated at 37 °C for 24 h. After 24 h, methylcellulose overlay was removed, cells were washed twice with PBS and fixed with 2% paraformaldehyde in PBS for 30 min at room temperature. Following fixation, plates were washed twice with PBS and foci were visualized on a fluorescence ELISPOT reader (CTL ImmunoSpot S6 Universal Analyzer) and enumerated using Viridot^29^. The neutralization titers were calculated as follows: 1 - (ratio of the mean number of foci in the presence of sera and foci at the highest dilution of respective sera sample). Each specimen was tested in two independent assays performed at different times. The FRNT-mNG_50_ titers were interpolated using a 4-parameter nonlinear regression in GraphPad Prism 8.4.3. Samples with an FRNT-mNG_50_ value that was below the limit of detection were plotted at 10. For these samples, this value was used in fold reduction calculations.

### Focus Reduction Neutralization Titer assay against the variants of concern

The wildtype infectious clone SARS-CoV-2 (icSARS-CoV-2), derived from the 2019-nCoV/USA_WA1/2020 strain, was propagated in VeroE6 cells (ATCC) and sequenced^28^. The RSA B.1.351 variant was isolated as previously described^30^. Our laboratory plaque-isolated the virus on VeroE6 cells followed by a single round of propagation on VeroE6 cells (MOI 0.05), aliquoted to generate a working stock and sequenced. Viral titers were determined by focus-forming assay on VeroE6 cells. Viral stocks were stored at −80°C until use.

FRNT assays were performed as previously described for the WT FRNT assay. The assay with each variant was performed simultaneously with WT controls. The samples were diluted at 3-fold in 8 serial dilutions using DMEM in duplicates with an initial dilution of 1:10 in a total volume of 60 μl. Serially diluted samples were incubated with an equal volume of SARS-CoV-2, WT or the variant, (100-200 foci per well) at 37° C for 1 hour in a round-bottomed 96-well culture plate. The Ab-virus mixture was then added to Vero cells and incubated at 37° C for 1 hour. Post-incubation, the Ab-virus mixture was removed and 100 μl of prewarmed 0.85% overlay was added to each well. Plates were incubated at 37°C for 24 hours. After 24 hours, methylcellulose overlay was removed, and cells were washed three times with PBS. Cells were then fixed with 2% paraformaldehyde in PBS (Electron Microscopy Sciences) for 30 minutes. Following fixation, plates were washed twice with PBS and 100 μl of permeabilization buffer (0.1% BSA, Saponin in PBS), was added to the fixed Vero cells for 20 minutes. Cells were incubated with an anti-SARS-CoV spike primary Ab directly conjugated to biotin (CR3022-biotin) for 1 hour at room temperature. Next, the cells were washed three times in PBS and avidin-HRP was added for 1 hour at room temperature followed by three washes in PBS. Foci were visualized using TrueBlue HRP substrate (KPL, # 5510-0050) and imaged on an ELISPOT reader (CTL).

### Intracellular cytokine staining assay

Antigen-specific T cell responses were measured using the ICS assay as described previously^31^. Live frozen PBMCs were revived, counted and resuspended at a density of 2 million live cells/ml in complete RPMI (RPMI supplemented with 10% FBS and antibiotics). The cells were rested overnight at 37°C in CO_2_ incubator. Next morning, the cells were counted again, resuspended at a density of 15 million/ml in complete RPMI and 100 μl of cell suspension containing 1.5 million cells was added to each well of a 96-well round-bottomed tissue culture plate. Each sample was treated with two conditions, no stimulation, and a peptide pool spanning the S protein at a concentration of 1 μg/ml of each peptide in the presence of 1 μg/ml of anti-CD28 (clone CD28.2, BD Biosciences) and anti-CD49d (clone 9F10, BD Biosciences) as well as anti-CXCR3 and anti-CXCR5. The peptides were custom synthesized to 90% purity using GenScript, a commercial vendor. All samples contained 0.5% v/v DMSO in total volume of 200 μl per well. The samples were incubated at 37°C in CO2 incubators for 2 h before addition of 10 μg/ml Brefeldin-A. The cells were incubated for an additional 4 h. The cells were washed with PBS and stained with Zombie UV fixable viability dye (Biolegend). The cells were washed with PBS containing 5% FCS, before the addition of surface Ab cocktail. The cells were stained for 20 min at 4°C in 100 μl volume. Subsequently, the cells were washed, fixed and permeabilized with cytofix/cytoperm buffer (BD Biosciences) for 20 minutes. The permeabilized cells were stained with ICS antibodies for 20 min at room temperature in 1X-perm/wash buffer (BD Biosciences). Cells were then washed twice with perm/wash buffer and once with staining buffer before acquisition using the BD Symphony Flow Cytometer and the associated BD FACS Diva software. All flow cytometry data were analyzed using Flowjo software v10 (TreeStar Inc.).

### Bead-based antigen arrays.

We used an existing bead-based autoantigen array, and a cytokine array with expanded content that was based on our recent COVID-19 studies^16^. A complete list of all antigens, vendors, and catalogue numbers can be found in Supplementary Tables 2 and 3. The “COVID-19 Autoantigen Array” included 55 commercial protein antigens associated with connective tissue diseases (CTDs) (Supplementary Table 2). The “COVID-19 Cytokine Array” comprised 58 proteins including cytokines, chemokines, growth factors, acute phase proteins, and cell surface proteins (Supplementary Table 3). Antigens were coupled to carboxylated magnetic beads (MagPlex-C, Luminex Corp.) such that each antigen was linked to beads with unique barcodes, as previously described^16,32,33^. Prototype human plasma samples derived from participants with autoimmune diseases with known reactivity patterns were purchased from ImmunoVision or were obtained from Stanford rheumatology clinics and had been characterized previously^16^. APS-1, PAP, or AMI serum samples were used for validation of ACA^16^. Serum samples were tested at 1:100 dilution in 0.05% PBS-Tween supplemented with 1% (w/v) bovine serum albumin (BSA). Bound antibody was detected using R-phycoerythrin (R-PE) conjugated Fcγ-specific goat anti-human IgG F(ab')2 fragment (Jackson ImmunoResearch) prior to analysis using a FlexMap3D™ instrument (Luminex Corp.). A minimum of 100 events per bead ID were counted, and samples were studied in duplicate. Binding events were displayed as Mean Fluorescence Intensity (MFI). All data analysis and statistics were performed using R and various R packages^34^. For normalization, average MFI values for “bare bead” IDs were subtracted from average MFI values for antigen-conjugated bead IDs.

### CyTOF analysis of whole blood samples

Fresh whole blood samples collected in sodium citrate cell preparation tubes (CPT) were fixed in proteomic stabilizer buffer. 270 μl of whole blood samples were mixed with 420 μl of PROT1 stabilizer (Smart tube Inc, San Carlos, CA), mixed and incubated at room temperature for 12 min and frozen at −80°C until processing. Fixed frozen cells were thawed by gentle resuspension in CSM (PBS supplemented with 2% BSA, 2 mM EDTA, and 0.1% sodium azide), washed twice with CSM. After thawing the stabilized blood samples, they were added to 1X Thaw-Lyse buffer (Smart Tube), incubated 10 min at RT, then centrifuged and resuspended again in Thaw-Lyse buffer. After another 10 min at RT, they were centrifuged and washed in 1 ml CSM. They were then permeabilized, barcoded and stained with pre-titrated intracellular antibody cocktail for 30 min at room temperature. Cells were then washed with CSM, stained with iridium-containing DNA intercalator (Fluidigm), washed with MilliQ water and acquired on Helios mass cytometer (Fluidigm) in MilliQ water supplemented with 1x EQ four element calibration beads (Fluidigm).

The FCS files were bead-normalized before data export. The data were processed for debarcoding in Flowjo software v10 (TreeStar Inc.). Briefly, the bead-normalized file was used to gate single cells based on DNA content and event length using FlowJo. The single cells were reimported and debarcoded using Helios software version 7.0.5189. The debarcoded samples were analyzed using FlowJo or R version 1.2.1335 for downstream tSNE analysis and visualization.

### CyTOF data analysis

High-dimensional analysis of phospho-CyTOF data was performed using an R based pipeline described in^35^. Briefly, the raw fcs files were imported into R and the data were transformed to normalize marker intensities using arcsinh with a cofactor of 5. For visualization, another transformation was applied that scales the expression of all values between 0 and 1 using percentiles as the boundary. Cell clustering was performed with 4,000 cells randomly selected from each sample using *FlowSom* and *ConsensusClusterPlus*. The transformed matrix was used as an input for *FlowSom* and cells were separated into 20 clusters. To obtain reproducible results (avoid random start), a seed was set for each clustering. The 20 clusters were manually annotated based on the lineage marker expression and were merged to produce the final clusters. The clusters were visualized in two-dimensional space using UMAP. The abundance of cell populations was determined using Plotabundance function. In parallel, the data were manually gated to identify 25 immune cell subpopulations that were not well-distinguished in UMAP and used for all quantitation purposes.

### Plasma protein profiling using multiplex Olink panel

We measured cytokines in plasma using Olink multiplex proximity extension assay (PEA) inflammation panel (Olink proteomics: www.olink.com) according to the manufacturer’s instructions and as described before (*41*). The PEA is a dual-recognition immunoassay, where two matched antibodies labelled with unique DNA oligonucleotides simultaneously bind to a target protein in solution. This brings the two antibodies into proximity, allowing their DNA oligonucleotides to hybridize, serving as template for a DNA polymerase- dependent extension step. This creates a double-stranded DNA “barcode” which is unique for the specific antigen and quantitatively proportional to the initial concentration of target protein. The hybridization and extension are immediately followed by PCR amplification and the amplicon is then finally quantified by microfluidic qPCR using Fluidigm BioMark HD system (Fluidigm Corporation. South San Francisco, California).

### Bulk Transcriptomics

Whole blood samples were collected in Paxgene tubes (BD Biosciences) and were frozen at −80°C until RNA isolation. RNA was isolated from each sample using the miRNeasy Mini kit (Qiagen) and 10 ng of total RNA was used as input for cDNA synthesis using the Clontech SMART-Seq v4 Ultra Low Input RNA kit (Takara Bio) according to the manufacturer’s instructions. Amplified cDNA was fragmented and appended with dual-indexed bar codes using the NexteraXT DNA Library Preparation kit (Illumina). Libraries were validated by capillary electrophoresis on an Agilent 4200 TapeStation, pooled at equimolar concentrations, and sequenced on an Illumina NovaSeq6000 at 100SR, yielding 20 million reads per sample. ENSEMBL IDs were filtered to remove low/non-expressed transcripts (0 reads in >50% of samples). Gene-level counts were created by averaging counts from all ENSEMBL IDs mapping to the same gene symbol (IDs mapping to multiple symbols were discarded), using the bioMart package.

### Bulk Transcriptomics analysis

Gene-level counts were filtered to remove those with a median expression less than 32. PCA was performed on baseline samples to identify outliers. Three samples were more than 1.5 standard deviations away from the mean and were removed from the analysis. RLE plots were generated with EDAseq^36^; samples with an RLE > 0.6 were removed from the analysis. Differential gene analysis was performed using DESeq2^37^ (v 1.26.0), incorporating participant id into the model to account for inter-participant bias. Genes were ranked by the Wald statistic as reported by DESeq2 for GSEA using the BTMs^19^. Per-participant fold changes were computed by dividing the DESeq2 normalized expression data for the day of interest by either day 0 (for day 1, day2, and day 7) or day 21 (for day 22, 28, and 42). The age of each participant was compared against the per-participant fold changes for day 22. The resulting correlation values were ranked by t-statistic and analyzed with GSEA using the BTMs to obtain the BTM correlates with age. The same method was employed to obtain BTM correlates with IFN-γ. IFN scores were computed by taking the per-participant mean fold change on day 22 of the unique set of genes present in the 5 interferon BTMs (M75, M111.1, M150, M127 and M68) that significantly correlated with day 22 IFN-γ fold change. Similarly, the per-participant M16 gene score was computed using average fold change on day 22 of the genes present in M16.

### CITE-seq

CITE-seq analysis of PBMCs were assayed exactly as described previously^4^. Briefly, live frozen PBMCs were thawed and 2x washed with RPMI supplemented with 10% FBS and 20 μg/mL DNAse I (Sigma Aldrich). DCs were enriched using the Dynabeads TM DC Enrichment Kit (Invitrogen, 11308D) according to manufacturer’s instructions with 3 – 4 million PBMCs as starting material. The enriched cells were mixed with total PBMCs at a ratio of 1:2 and mixed cells were stained with a cocktail of TotalSeq-A antibodies in PBS supplemented with 5% FBS, 2 mM EDTA, and 5 mg/mL human IgG **(Extended Data Table XX)**, washed twice with PBS supplemented with 5% FBS, and 2 mM EDTA, and resuspended in PBS supplemented with 1% BSA (Miltenyi), and 0.5 U/μL RNase Inhibitor (Sigma Aldrich). About 9,000 cells were targeted for each experiment.

Cells were mixed with the reverse transcription mix and subjected to partitioning along with the Chromium gel-beads using the 10X Chromium system to generate the Gel-Bead in Emulsions (GEMs) using the 3' V3 chemistry (10X Genomics, Pleasanton, CA). The RT reaction was conducted in the C1000 touch PCR instrument (BioRad). Barcoded cDNA was extracted from the GEMs by Post-GEM RT-cleanup and amplified for 12 cycles. Prior to amplification the cDNA amplification mix was spiked in with ADT additive primer (0.2 μM stock) in order to amplify the antibody barcodes. Amplified cDNA was subjected to 0.6x SPRI beads cleanup (Beckman, B23318). Amplified antibody barcodes were recovered from the supernatant and were processed to generate TotalSeq-A libraries as instructed by the manufacturer (BioLegend, TotalSeq-A antibodies with 10x Single Cell 3' Reagent Kit v3 3.1 protocol). The rest of the amplified cDNA was subjected to enzymatic fragmentation, end-repair, A tailing, adapter ligation and 10X specific sample indexing as per manufacturer’s protocol. Libraries were quantified using Bioanalyzer (Agilent) analysis.

10x Genomics scRNA-Seq and TotalSeq-A libraries were pooled and sequenced on an Illumina HiSeq 4000 using the recommended sequencing read lengths of 28 bp (Read 1), 8 bp (i7IndexRead), and 91bp (Read2). CellRangerv3.1.0 (10xGenomics) was used to demultiplex raw sequencing data and quantitate transcript levels against the 10x Genomics GRCh38 reference v3.0.0.

### CITE-Seq analysis

10x Genomics scRNA-Seq and TotalSeq-A libraries were pooled and sequenced on a Novaseq S4. Cell Ranger v3.1.0 (10x Genomics) was used to quantitate transcript levels against the 10x Genomics GRCh38 reference (v3.0.0.) Raw count data was filtered to remove cells with a mitochondrial RNA fraction greater than 20% of total RNA counts per cell, cells with fewer than 100 unique features, or cells with fewer than 200 total reads. The filtered count matrix was used to create a Seurat^38^ (v 3.1.4) object. Filtered read counts were scaled by a factor of 10,000 and log transformed. The antibody-derived tag matrix was normalized per feature using center log normalization. Doublets were identified with scds^39^ (v 1.2.0); cells with a doublet score in the top decile were removed. The remaining 242,479 cells were processed with the default Seurat pipeline. Specifically, the most variable 2000 RNA features were used to perform PCA on the log-transformed counts. The first 25 principle components were used further downstream analyses, including clustering and UMAP projections. Clusters were identified with Seurat SNN graph construction followed by Louvain community detection on the resultant graph with a resolution of 0.2, yielding 18 clusters. Differential expression across timepoints was calculated with MAST^40^ (v 1.12.0) to account for inter-participant heterogeneity.

Pseudobulk profiles were constructed by taking the average expression across all cells in each participant, per day. When computing fold changes across timepoints, each participant’s pseudobulk profile was compared to their baseline profile to reduce participant-specific biases. To calculate the impact of removing a cluster, each cluster across all timepoints was iteratively removed and resulting fold-changes were recomputed.

C8 was re-embedded and reclustered with UMAP and Louvain community detection, respectively. Distances from each sub-cluster to the other clusters was calculated as the Euclidean distance between the average expression of all genes of each cluster.

Complexheatmap (v. 2.2.0) was used for all heatmaps. All analysis was performed in R (v 3.6.3)

## Extended Data

**Extended Data Table 1.** Participant demographics

Variable	Total N = 56 N (%)
**Age (median [range])**	36 (19-79)
**Sex**	
Female	28 (50)
Male	28 (50)
**Race**	
White	27 (48.2)
Asian	22 (39.3)
Black	4 (7.1)
Native American	1 (1.8)
Other	2 (3.6)
**Ethnicity**	
Not of Hispanic, Latinx, or Spanish origin	54 (96.4)
Hispanic, Latinx, Spanish origin	2 (3.6)
**Comorbidities**	
Lung disease	8 (14.2)
Diabetes mellitus	2 (3.6)
Hypertension	1 (1.8)
Cardiovascular	2 (3.6)
Liver disease	0 (0)
Renal diseases	1 (1.8)
Cancer	1 (1.8)
Hematological disorder	1 (1.8)
Pregnancy	0 (0)
Neurological	4 (7.1)
HIV	0 (0)
Solid organ transplant recipient	0 (0)
Bone marrow transplant recipient	0 (0)
Hyperlipidemia	1 (1.8)
Gastrointestinal	4 (7.1)
Psychiatric	1 (1.8)
Genitourinary disorder	3 (5.3)
Hypersensitivity	6 (10.7)
Autoimmune disorder	4 (7.1)
**Confirmed SARS-CoV-2 infection**	4 (7.1)
Time from SARS-CoV-2 infection to baseline visit in days (median [range])	180 (48-270)

**Extended Data Table 2.** Vaccine side effects and symptoms

Variable	Total N=56 N (%)	Variable	Total N=56 N (%)	p-value
**First Dose**		**Second Dose**		
None	11 (19.6)	None	5 (8.9)	0.177
Fever	2 (3.6)	Fever	7 (12.5)	0.154
Site tenderness	39 (69.6)	Site Tenderness	40 (71.4)	0.881
Site swelling	6 (10.7)	Site Swelling	16 (28.6)	0.029
Site redness	4 (7.1)	Site Redness	14 (25)	0.018
Muscle aches	15 (26.8)	Muscle aches	29 (51.8)	0.015
Fatigue	15 (26.8)	Fatigue	21 (37.5)	0.28
Headache	10 (17.9)	Headache	17 (30.4)	0.167
Chills	2 (3.6)	Chills	10 (17.9)	0.03
Joint pain	1 (1.8)	Joint Pain	4 (7.1)	0.349
Nausea	1 (1.8)	Nausea	2 (3.6)	0.49
Difficulty breathing	1 (1.8)	Difficulty Breathing	1 (1.8)	1
Hives	0 (0)	Hives	1 (1.8)	0.993
Swelling	0 (0)	Swelling	2 (3.6)	0.468
Tachycardia	0 (0)	Tachycardia	1 (1.8)	0.993
Rash	0 (0)	Rash	2 (3.6)	0.468
Dizziness	2 (3.6)	Dizziness	5 (8.9)	0.317
Sweating	2 (3.6)	Sweating	1 (1.8)	1
Brain fog	1 (1.8)	Brain Fog	0 (0)	0.993
Loss of appetite	1 (1.8)	Loss of appetite	0 (0)	0.993
Rhinitis	1 (1.8)	Rhinitis	0 (0)	0.993
Paresthesia	1 (1.8)	Paresthesia	1 (1.8)	1
Diarrhea	1 (1.8)	Diarrhea	0 (0)	0.993
Vertigo	0 (0)	Vertigo	1 (1.8)	0.993
**Site of injection**		**Site of injection**		
Right arm	5 (8.9)	Right arm	48 (85.7)	
Left arm	51 (91.1)	Left arm	48 (85.7)	

**Extended Data Table 3.** CyTOF panel

Conjugate	Target
89Y	CD66b
113In	CD57
141Pr	HLA-DR
142Nd	CD19
143Nd	CD127
144Nd	IL4
145Nd	CD4
146Nd	IgD
147Sm	CD20
148Nd	CD34
149Sm	pSTAT6
150Nd	pStat5 (Y694)
151Eu	CD123 (IL-3R)
152Sm	Siglec-9
153Eu	pStat1 (Y701)
154Sm	H3K27ac
155Gd	CD27
156Gd	CD45
157Gd	CD25
158Gd	pStat3 (Y705)
159Tb	CD11c
160Gd	CD14
161Dy	Ki67
162Dy	CD69
163Dy	TCRgd
164Dy	Arginase-1
165Ho	pCREB (S133)
166Er	CD16
167Er	CD38
168Er	CD8a
169Tm	CD45RA
170Er	CD3
171Yb	Granzyme-B
172Yb	CD15
174Yb	IFN-γ
175Lu	pS6
176Yb	CD56

**Extended Data Table 4.** Vaccine meta-analysis datasets

Vaccine	Pathogen	Vaccine Type	Adjuvant/ Vector	Timepoints used	N	GEO/ ImmPort
BNT162b2	SARS-CoV-2	mRNA	mRNA-LNP	0,1,7,21,22,28	31	
IIV	Seasonal Influenza	Inactivated	None	0,1,7	19	GSE78813/SDY56
VZV	Varicella zoster	Live attenuated	VZV	0,1,7	31	GSE79396/SDY984
YF17D	Yellow fever	Live attenuated	YF17D	0,1,7	25	GSE13486/SDY1264
rVSV-ZEBOV	Ebola	Recombinant viral vector	VSV	0,1,7	7	GSE97590/SDY1373
MRKAd5/HIV	HIV	Recombinant viral vector	Ad5	0,1,7	10	GSE22768/SDY1291
H5N1+AS03	H5N1 Influenza	Inactivated	AS03	0,1,7, 21,22,28	33	GSE102012
MPSV4	Meningococcus	Polysaccharide	None	0,7	13	GSE52245/SDY1260
MCV4	Meningococcus	Conjugate	None	0,7	17	GSE52245/SDY1260

## Supplementary Material

Supplement 1

Supplement 2

Supplement 3

## Data Availability

CITE-seq and bulk RNA data are publicly accessible in the Gene Expression Omnibus under accession numbers GSE171964 and GSE169159, respectively.
